# Validation of the Lithuanian Version of the Coach-Created Empowering and Disempowering Motivational Climate Questionnaire (EDMCQ-C)

**DOI:** 10.3390/ijerph17103487

**Published:** 2020-05-16

**Authors:** Saulius Sukys, Enrika Kromerova-Dubinskiene, Paul R. Appleton

**Affiliations:** 1 Department of Physical and Social Education, Lithuanian Sports University, Sporto 6, LT-44221 Kaunas, Lithuania; enrika1991@gmail.com; 2 School of Sport, Exercise and Rehabilitation Sciences, University of Birmingham, Edgbaston, Birmingham B15 2TT, UK; p.appleton@bham.ac.uk

**Keywords:** EDMCQ-C, adolescent athletes, discriminant, convergent, predictive validity

## Abstract

Based on Duda’s conceptualization of the motivational climate, the Empowering and Disempowering Motivational Climate Questionnaire-Coach (EDMCQ-C) is a recently developed scale that assesses junior athletes’ perception of the social environmental dimensions proposed by achievement goal theory and self-determination theory. The goal of the current investigation was to evaluate the Lithuanian translation of the EDMCQ-C and more broadly extend the validity and reliability of this questionnaire in sport participants. 712 adolescents from different sport teams in Lithuanian were the participants in this study. Exploratory structural equation modelling provided an acceptable fit of a two-factor model (i.e., empowering and disempowering) of EDMCQ-C. Reliability analysis revealed good levels of internal consistency for the empowering and disempowering climate factors. Discriminant validity was confirmed by a negative correlation between empowering and disempowering climate subscales. Correlations between empowering and disempowering subscales with values, motivation and self-esteem constructs demonstrate convergent validity. Associations between the climate dimensions and prosocial and antisocial behaviour in sport demonstrate predictive validity of EDMCQ-C. The evidence from this study suggests the Lithuanian version of EDMCQ-C is a promising scale for the assessment of athletes’ perceptions of the empowering and disempowering features of the motivational climate created by their coach.

## 1. Introduction

Participation in sport is one of the most widespread ways of self-realization and a phenomenal context contributing to the development of human physical abilities [1] and social skills [2], as well as impacting attitude and motivation [3] and improving one’s health and well-being [4]. Among other things, sport activities can provide positive emotions, encourage self-esteem, strengthen socialization and help not only to establish but also to maintain long-lasting interpersonal relationships [5,6]. However, not all sport participants experience these positive outcomes and fail to continue their engagement in sport over the long-term [7,8,9]. While the decision to quit a sport can be influenced by many reasons, it is argued that enjoyment, perceptions of competence [9,10], social pressure, competing priorities and physical factors [9] are the most important in evaluating athletic experience and making decisions about the future. However, by its very nature, sport has a broad social context and its participants interact and can influence each other’s experiences and motivation. Studies show that personal motivation is an essential component in understanding and predicting various experiences and outcomes in sport [11].

The coach is often considered to be one of the most influential factors in determining whether athletes have a positive or negative experience in sport [12,13,14]. In particular, there is substantial evidence that the psychosocial environment or, in other words, the motivational climate, created by the coach can have a significant effect on an athlete’s perceptions, emotion and behaviour [15,16,17].

It is emphasized [18] that, when conceptualizing (and subsequently measuring) the coach-created motivational climate, it is important to consider achievement goal theory (AGT) [19] and self-determination theory (SDT) [20]. In the field of sport, the principles of AGT and SDT are very widely applied because they consider the broad range of motivational strategies of coaches and how these strategies impact on athletes [21,22].

AGT argues that the motivational climate captures what the coach says, how he/she acts and structures the psychosocial environment with reference to competence [23]. More specifically, AGT proposes that via the motivational climate created by the coach, athletes can view competence as task-involving and/or ego-involving. With a task-involving view, athletes feel competent when they exert effort, master skills and demonstrate personal development [19]. A task-involving view of competence is thought to develop within a task-involved motivational climate in which the coach values effort and learning, encourages cooperative learning between teammates and all participants have a significant role within the session and team/club [24,25].

In contrast, an ego-involving view is characterized by the athlete defining competence as outperforming his/her teammates and/or opponents with minimal-to-no effort [19,26]. An ego-involving view of competence is assumed in AGT to emerge in a coach-created motivational climate that encourages rivalry among team members, punishes mistakes and reinforces comparative ability [24,25]. Duda and Balaguer [27] emphasized that, “perceptions of a task-involving environment cultivated by the coach are linked to indices of an adaptive achievement pattern and more positive cognitive and emotional responses among athletes” (p. 122). In contrast, an ego-involving coach created motivational climate has been linked to indices of “a maladaptive achievement pattern and more negative cognitive and emotional responses” (p. 123 [27]).

A basic assumption of SDT is that the degree to which an individual’s motivation is self-determined and experiences optimal (or reduced) functioning is partly determined by the extent to which the social psychological environment strengthens or frustrates the satisfaction of three congenital human psychological needs—namely, competence, autonomy and relatedness [28]. In sport, athletes that experience satisfaction of their psychological needs are more likely to experience autonomous reasons for engaging in their activity, an in turn adaptive and healthy engagement which is conducive to sustained participation and positive well-being [22]. Conversely, the frustration and/or dissatisfaction of an athletes’ basic psychological needs is associated with lower levels of self-determined motivation and more controlled reasons for engagement (e.g., extrinsic incentives, penalties or out of feeling of quilt and pressure) and subsequently compromised health and dropout of sport [29,30,31,32,33].

The social contextual factors within SDT that are considered to satisfy an athlete’s psychological needs include autonomy and social support [23,34]. In an autonomy-supportive environment, the coach takes into account the interests and goals of athletes, their feelings are valued and they are included in decision-making [13]. In a socially supportive environment, the coach ensures athletes feel that they are cared for and valued as people (and not just sport participants) [13,35]. SDT also consider the climate dimensions that frustrate (and result in low satisfaction of) an athlete’s psychological needs. One dimension that has received attention in previous research in sport is a controlling climate, which is evident when a coach pressures, intimidates and coerces his/her athletes [36].

Previous research has examined facets of the coach-created motivational climate from either an AGT or SDT perspective or by simultaneously examining the targeted climate facets emphasised within both theories for example, [35,37,38]. A specific focus of research that has adopted the latter perspective has been to identify and understand the motivational mechanisms (in particular, basic psychological needs) that may account for hypothesised relationships between features of empowering and disempowering motivational climates described above and cognitions, affect and behaviour experienced in sport. This research has demonstrated that empowering features of the coach-created motivational climate (namely, task-involving, autonomy-support and socially supportive climates) positively predicted self-reported feelings of competence, autonomy and relatedness in young athletes from the UK [35]. Previous research (e.g., [37]) has also confirmed that facets of the motivational climate proposed within AGT and SDT vary in their relationships with and predict unique variance in, basic psychological needs when the climate dimensions are examined simultaneously. In a recent study with dancers, for example, a task-involving climate (created by dance instructors) positively predicted all three of the psychological needs proposed by SDT albeit the strongest path was with the competence need [37]. Moreover, Quested and Duda‘s findings revealed that an autonomy-supportive climate was a stronger predictor compared to a task-involving climate with regards to dancers‘ feelings of autonomy and relatedness. Finally, an examination of the relationships between the climate dimensions proposed within SDT and AGT in previous research in sport also confirms interdependent but not perfect associations (i.e., r < 1). Overall, these findings suggest the motivational climate dimensions proposed by AGT and SDT are inter-related but when considered simultaneously, each dimension holds important implications for athletes’ basic psychological needs [39].

Although much research has established the importance of examining the aforementioned climate dimensions from an AGT or SDT perspective in sport, Duda and colleagues [39] proposed that “a fuller understanding of the potential impact and determinants of the coach-created motivational climate should emerge when the climate dimensions emphasized in AGT and SDT are considered simultaneously” (*p*. 55). From this multiple theory approach, the motivational climate can be more or less empowering and/or disempowering [18]. An empowering coach-created motivational climate is characterized by task-involving, autonomy supportive and socially supportive features and a disempowering coach-created climate is marked by ego-involving and controlling characteristics.

Based on Duda’s [18] hierarchical and multidimensional model, the Empowering and Disempowering Motivational Climate Questionnaire (EDMCQ) has recently been developed as a measure of young athletes’ perceptions of their coaches’ motivation-related strategies [39]. Across a series of studies with junior sport participants in England, Appleton and colleagues tested a number of alternative models of the EDMCQ’s structure. The initial findings from Appleton et al.’s study revealed that a bi-factor exploratory structural equation model solution resulted in the best fitting solution, albeit the solution was not perfect. In particular, many autonomy-supportive and some controlling and socially supportive items failed to load significantly on their intended factor and demonstrated elevated and significant factor loadings on non-intended dimensions (i.e., controlling items loaded onto the ego-involving dimension; socially-supportive and autonomy-support items loads onto the task-involving dimension).

The findings reported by Appleton et al. [39] indicate that, in its current format, two composite factors may better represent the structure of the EDMCQ whereby task-involving, autonomy-support and socially-supportive items load onto an empowering factor and ego-involving and controlling items load onto a disempowering factor. This two-dimensional model was recently supported in a study testing the EDMCQ’s structure using Welsh secondary school pupils’ perceptions of the motivational climate created by their PE teachers [40].

Although further work is required to resolve the aforementioned issues associated with the scale’s factor structure, the EDMCQ-C has been successfully used in previous research where a positive relationship between athletes’ perceptions of a coach-created empowering climate and autonomous motivation [41,42], sport-related enjoyment [43] and health and optimal functioning [15] have emerged, offering initial support for the predictive validity of the scale. However, to date, the majority of published studies examining the psychometric properties of the EDMCQ-C have been conducted with athletes from England (for an exception [44,45]) and thus additional research is required to test the validity and reliability of the scale using data from sport participants in other countries.

Building upon the initial work of Duda [18], Appleton and colleagues [39], the main purpose of this study was to investigate the validity and reliability of an adapted version of the EDMCQ-C translated into Lithuanian in adolescent sport participants. Further examination of the validity and reliability of the EDMCQ-C will make an important contribution to establishing the psychometrics of the scale. Moreover, a psychometrically sound measure of empowering and disempowering strategies employed by coaches and perceived by their athletes would be an important tool for furthering research on the motivational climate in Lithuania. Based on the findings of Milton et al. in PE, we provided an initial test of a two-factor model of the EDMCQ-C’s structure. With a view of providing further evidence for the validity of the scale, we also examined the discriminant and convergent validity, as well as predictive validity on the EDMCQ-C.

For the discriminant and convergent validity, we studied relationships among the composite empowering and disempowering subscales and also relationship with other variables. In the current study, we expected a weak negative association between empowering and disempowering subscales. Based on Duda’s framework and previous research that has investigated empowering and disempowering features of the motivational climate for example, [15,42,43,46], we expected positive correlations between an empowering climate and athletes’ moral values, intrinsic motivation and self-esteem. We also hypothesised that an empowering climate would be negatively correlated with status sport values and amotivation. In contrast, we hypothesised that a disempowering motivational climate would be negatively correlated with athletes’ moral values, intrinsic motivational and self-esteem but positively associated with status values and amotivation. For the predictive validity, we expected that empowering climate will be positive predictor of prosocial behaviour and a negative predictor of antisocial behaviour. We also expected athletes’ perceptions of the disempowering climate to negatively predict prosocial behaviour and positively predictor antisocial behaviour.

## 2. Materials and Methods

### 2.1. Recruitment and Study Participants

Participants for this study were recruited by applying convenience and cluster random sampling from different sport teams from the middle of Lithuania. Inclusion criteria included (a) aged between 12 and 17 years of age; (b) participants had at least one year of participation in their current sport; (c) able to speak and understand Lithuanian. A total of 712 adolescents participated in this study. All participants were competitive at the national level. Table 1 presents the demographics of the study sample.

Data were collected at the three separate times and formed the three samples. The data provided by the participants in sample one, which was formed by applying convenience sampling, was used to examine the face validity of the EDMCQ-C translated into Lithuanian. Following corrections, the data provided by participants in group two, which was formed by applying convenience sampling, was used to test the factor structure of the questionnaire. Finally, data provided by group three was used for cross-validation of the structure of the questionnaire. Also, the discriminant, convergent and predictive validity of EDMCQ-C was examined using data provided by group three. Participants for this group were selected using a cluster random sampling. Assuming a 95% confidence level and ± 5% confidence interval, the recommended sample size was no less than 370 adolescents. Athletes in all three samples completed the EDMCQ-C and athletes in sample three also completed a number of other questionnaires (values in sport, sport motivation, self-esteem and prosocial and antisocial behaviour in sport).

#### 2.1.1. Group One

The group was comprised of 72 males and 53 females aged between 13 and 16 years old (N = 125; age mean 14.88, SD = 0.76). Participants were basketball and volleyball players who participated in their current sport on average 6.22 years (SD = 1.55).

#### 2.1.2. Group Two

The sample (N = 202) comprised 159 males and 43 females aged between 13 and 16 years old (M = 14.5; SD = 1.10). The athletes participated in basketball (n = 80), football (n = 57), volleyball (n = 29) and track and field (n = 36) sports. The mean number of seasons the participants had been playing their current sport was 4.10 (SD = 2.46).

#### 2.1.3. Group Three

The sample (N = 385) comprised 227 males and 158 females aged between 13 and 16 years old (M = 14.2; SD = 1.11). The athletes participated in basketball (n = 151), football (n = 55), volleyball (n = 108) and track and field (n = 71) sports. The mean number of seasons the participants had been playing their current sport was 3.95 (SD = 2.44). Participants in group three were not from the same teams as participants in groups one and two.

### 2.2. Measures

#### 2.2.1. Perceptions of the Coach-Created Motivational Climate

In order to achieve a Lithuanian version of the EDMCQ-C, we used a forward and backtranslation procedure [47]. Firstly, the scale text was translated by three Lithuanian native speakers who had higher education in English language. One of the translators also had a PhD in sport science. In translating the scale text, emphasis was given to the conceptual equivalent of words and phrases (instead of providing a literal translation). Next, two authors of this paper discussed any disagreement in the translation until they reached agreement. Finally, the scale text was backward translated from Lithuanian to English by two English language professionals (who were not the same individuals providing the first translation from English to Lithuanian) and any discrepancies were discussed by the authors of this research until agreement was reached. This resulted in a 34 item Lithuanian version of the EDMCQ-C.

Participants from group one subsequently rated the 34 items on a 5-point scale (e.g., 1 = absolutely unclear, 5 = completely clear) to determine the extent to which the translation was understood by junior sport participants. Participants were also asked to note any changes they would make to the questionnaire items and formatting. Athletes reported that it was a little difficult to understand the word “pratybos” (training). This finding was unsurprising given there are two words in Lithuania which represent training (“treniruotė,” “pratybos”), with the latter more common in scientific language (and less common in the everyday language of youth sport participants). It should be noted that only two items including the phrase pratybos were evaluated as unclear (i.e., a score of two on the Likert Scale) resulting in a change in the wording. The Lithuanian version of the EDMCQ-C items can be seen in the Supplemental Material.

Following these changes, the Lithuanian EDMCQ-C was used with participants from group two and three. Following the stem “So far this season,” participants responded to 17 items assessing their perception of empowering features of the coach-created motivational climate (e.g., “My coach gave players choices and options,” “My coach made sure everyone had an important role on the team”) and 17 items assessing their perceptions of disempowering features of the coach-created motivational climate (e.g., “My coach had his or her favourite players,” “My coach was less accepting of players if they disappointed him or her”). Participants responded to items on a 5-point scale (i.e., 1 = strongly disagree, 5 = strongly agree).

#### 2.2.2. Values in Sport

The Youth Sport Values Questionnaire-2 [48] was used to measure important values in sports activities. Participants from group three responded to 13 items, using seven response options from especially important to me (5) to contrary to what I believe (–1). The thirteen statements can be grouped into moral (5 items, for example, “I always play properly”), competence (4 items, for example, “I use my skills well”) and status (4 items, for example, “I show that I am better than others”) values. The Lithuanian version of this scale was validated in previous studies with student athletes [49]. Lithuania athletes’ scores on this scale have demonstrated good internal consistency with alpha coefficients ranging from 0.70 to 0.85 [49].

#### 2.2.3. Sport Motivation

The Sport Motivation Scale (SMS) [50] was used to measure participants’ intrinsic motivation and amotivation. Response option range from 1 (does not correspond at all) to 7 (corresponds exactly). Although a revised version of this scale has been developed (SMS-II) [51], we used the original SMS as it has been previously validated in Lithuanian [52] with alpha coefficients ranging from 0.65 to 0.80. More specifically, in this study we used intrinsic motivation subscale (12 items assessing intrinsic motivation to know, to accomplish and to experience stimulation) and amotivation subscale (4 items). Support for combining the three types of intrinsic motivation into single measure has previously been provided in sport literature [53,54].

#### 2.2.4. Self-Esteem

Self-esteem was measured using Rosenberg’s [55] 10-item self-esteem scale with five positively and five negatively worded items. All items (e.g., “I feel that I have a number of good qualities,” “I feel I do not have much to be proud of”) were answered using a 4-point Likert scale format ranging from 1 = strongly disagree to 4 = strongly agree. Five items were reverse scored. This scale has been used in previous studies in Lithuania including research with sport samples [56] with good internal consistency alpha coefficient (e.g., 0.86).

#### 2.2.5. Prosocial and Antisocial Behaviour in Sport

Prosocial and Antisocial Behaviour in Sport Scale (PABSS) [57] was used to measure athletes’ prosocial and antisocial behaviour in sport. Participants were presented with 20 items and were asked to report how often they had engaged in each behaviour this season on a Likert scale anchored by 1 = never and 5 = very often. The PABSS consists of four subscales that measure antisocial behaviour toward opponents and toward teammates (e.g., “Tried to injure an opponent,” “Physically intimidated an opponent”) and prosocial behaviour toward opponents and teammates (e.g., “Encouraged a teammate,” “Helped an injured opponent”). The Lithuanian version of this scale was validated in previous studies with athletes’ scores demonstrating good internal consistency with alpha coefficients ranging from 0.79 to 0.85 [49]. In the current study, we employed a composite prosocial behaviour score and antisocial behaviour score.

### 2.3. Procedures

Prior to commencement of the study, approval was received from the University Ethics Committee of the first and third authors (No SMTEK-12). Initial contact was made with the representatives of youth teams/clubs to obtain their permission to approach athletes regarding the study. Written parental informal consent also was obtained before the involvement of children. The athletes also received verbal information regarding the aim of the study and their voluntary participation. Athletes completed the questionnaire after a training session in the presence of research assistance. Completion of the questionnaires ranged from 15 to 25 min. Data were collected between October 2017 and April 2018.

### 2.4. Validity Analysis

In this study first, factorial structure of the Lithuanian version of EDMCQ-C was evaluated. Next, discriminant and convergent validity were assessed. Discriminant validity entails the evaluation of measures against each other, is evident when a set of variables presumed to measure different constructs are not too highly interrelated [58] and was examined by considering the relationship among the composite empowering and disempowering subscales. Convergent validity refers to the degree to which a measure is associated with theoretically similar constructs [59]. Convergent validity was investigated by examining whether the athletes’ perceptions of empowering and disempowering climates were related to their values in sport, intrinsic motivation and amotivation and self-esteem. Finally, predictive validity was assessed. Predictive validity is one of two subcomponents of criterion-related validity [60]. Predictive validity concerns the operationalization ability of a variable to predict something that theoretically it should be able to predict. In the present study, we theorize that a measure of EDMSQ-C should be able to predict moral behaviour of athletes.

### 2.5. Data Analysis

#### 2.5.1. Descriptive Statistics

Data were analysed using Statistical Package for the Social Sciences (SPSS) 23.0 and Mplus 6. Preliminary data screening was conducted to check for missing values, outliers and normality. There were no missing data. Then we screened data for outliers using Z-scores. No extreme outliers were detected (i.e., values higher or lower than three SDs from the mean) [61]. Finally, the distribution of the data was acceptable because the skewness and kurtosis values were between ± 2 for all variables [61]. Descriptive analysis also included means, standard deviations and range values.

#### 2.5.2. Factorial Structure Evaluation

The factor structure of Lithuanian version of EDMCQC was tested using Exploratory Structural Equation Modelling (ESEM) [62,63]. ESEM is an analysis that incorporates the advantages of Exploratory (EFA) and Confirmatory (CFA) factor analysis. Initial validation of EDMCQ-C revealed better fit to the data for the ESEM solutions compared the CFA related structures [39]. Assessing model fit involved examining the comparative fit index (CFI) [64], the (robust) Tucker-Lewis index (TLI) [65] and the (robust) root mean square error of approximation (RMSEA) [66] with its 90% confidence interval. CFI and TLI values > 0.95 and RMSEA values < 0.06 were considered as indicators of excellent fit [67] and CFI and TLI values > 0.90 and RMSEA< 0.08 were considered as indicators of acceptable fit [68]. Data for ESEM analyses were treated as categorical.

#### 2.5.3. Reliability Analysis

For the reliability analysis, Cronbach’s alpha coefficients were calculated. Based on previous studies examining the psychometrics of the EDMCQ-C [39,44], alphas of > 0.80 were deemed acceptable.

#### 2.5.4. Discriminant and Convergent Validity

Discriminant and convergent validity were tested via Pearson correlation coefficients between the EDMCQ-C (LT) and subscale scores of moral values in sport, intrinsic motivation and amotivation in sport and self-esteem.

#### 2.5.5. Predictive Validity

For predictive validity, two multiple regression analyses were conducted, one each for prosocial and antisocial behaviour. In both analyses empowering and disempowering climate were entered as predictive variables. Prosocial behaviour was entered as dependent variable in the first and antisocial behaviour in the second analyses. Assumptions for multiple regression were evaluated before computing the models. Homoscedasticity was verified through visual examination of the graph of standardized residuals against standardized predicted values. The absence of multicollinearity was tested through the variance inflation factor (VIF) and Tolerance, the absence of autocorrelation was verified through the Durbin–Watson test. For all statistical analysis, we set statistical significance at *p* < 0.05.

## 3. Results

Using data from group two, the ESEM of the two factor EDMCQ-C model revealed acceptable fit of the data—CFI = 0.92, TLI = 0.91, RMSEA = 0.06 (90% CI = 0.05 to 0.06). The analysis supported a two-factor model with 17 empowering (with standardized factor loading ranged between 0.47–0.82) and 17 disempowering (with standardized factor loading ranged between 0.37–0.77) items (Table 2). Table 2 shows that four empowering climate items significantly and positively loaded onto the disempowering factor but with lower loading value (0.115 to 0.181) compared to the loading on the empowering factor. Also, one disempowering item significantly and positively loaded on to the empowering factor but with a loading value 0.168.

Data from group three were employed to cross-validate the two factor EDMCQ-C model. Regarding the ESEM, the data fit was acceptable—CFI = 0.94, TLI = 0.93, RMSEA = 0.06 (90% CI = 0.05 to 0.06). Result analysis showed the two-factor model with 17 empowering (with standardized factor loading ranged between 0.51–0.78) and 17 disempowering (with standardized factor loading ranged between 0.38–0.75) items (Table 3) loading onto their intended factor. Table 3 shows that three empowering climate items significantly and positively loaded on to the disempowering factor (0.123 to 0.163) and two disempowering items significantly and positively loaded (0.119 and 0.168) on to the empowering factor, albeit these items had stronger loading on their intended factor.

Reliability analysis with group two data revealed good levels of internal consistency, with a Cronbach alpha of 0.86 for the empowering climate factor and 0.89 for the disempowering climate factor. Reliability analysis with group three data also revealed good levels of internal consistency, with a Cronbach alpha of 0.87 for both factors. Descriptive statistics showed that the mean for empowering in group two was 4.34 (SD = 0.43) and group three 4.31 (SD = 0.45) and disempowering mean score of 2.68 (SD = 0.65) and 2.69 (SD = 0.65) for group two and three, respectively.

Aiming to provide further evidence for the validity of the EDMCQ-C, Pearson’s correlation was conducted with the third sample to examine discriminant and convergent validity. Regarding discriminant validity of EDMCQ-C, a negative weak correlation between those two climate dimensions emerged in groups two (r = −0.18, *p* < 0.01) and group three (r = −0.23, *p* < 0.01).

Assessing the convergent validity of EDMCQ-C involved examining the correlations between empowering and disempowering climate subscales with values, intrinsic motivation and amotivation and self-esteem scores. As can be seen in Table 4, empowering climate scores were positively related to moral and competence values, intrinsic motivation and athlete’s self-esteem. Empowering climate scores were also positively related with status values but this relationship was weak. Finally, empowering climate scores were negatively correlated with amotivation. Convergent validity was also supported by a negative association between disempowering climate scores and athletes’ self-esteem and positive correlations with status value and amotivation (Table 4). However, the correlations between disempowering climate scores with moral and competence values and intrinsic motivation, were non-significant.

To assessing predictive validity, we conducted two regression analyses to test whether empowering and disempowering climate predicted the moral behaviour of athletes. The results of the analyses (Table 5) indicated that empowering climate scores positively predicted prosocial behaviour of athletes (β = 0.26, *p* < 0.001). Analyses showed that disempowering climate emerged as a positive predictor for antisocial behaviour (β = 0.30, *p* < 0.001). Assumptions for multiple regression were met. An analysis of standard residuals was carried out, which showed that the data contained no outliers, identified as scores more than 3.29 SD from the mean. Multicollinearity was not a concern, as VIF values were lower than 10 and Tolerance higher than 0.1. The data also met assumption independent error as Durbin-Watson values were close to 2.

## 4. Discussion

Development of the EDMCQ-C [39] was an important step in assessing junior athletes’ perception of the social environmental dimensions proposed by achievement goal and self-determination theories. Originally developed using data from samples of junior athletes in England, the analysis of the EDMCQ-C’s psychometric properties in different cultural contexts is an important line of research that will enable comparative studies analysing empowering and disempowering features of the coach-created motivational climate across countries. The development of a valid and reliable EDMCQ-C in a range of languages will also enable researchers to evaluated interventions aimed at optimizing the coach-created motivational climate and subsequent impact on coaching behaviours and athletes. Thus, the aim of this study was to investigate the validity and reliability of the EDMCQ-C in a sample of Lithuanian adolescent sport participants.

### 4.1. Factorial Structure of the EDMCQ-C

The first step in the validation of the Lithuania version of the EDMCQ-C was the evaluation of the scale’s factorial structure. Although the original factor structure of the EDMCQ-C tested by Appleton et al. [39] in youth sport was consistent with Duda’s multidimensional and hierarchical conceptualization of the motivational climate (i.e., 5 lower-order dimensions, 2 higher-order dimensions), this initial test revealed a number of items loaded more strongly on non-intended factors. In response, Milton et al. [40] recently offered support for a two-factor lower-order model in a sample of PE students with task-involving, autonomy-supportive and socially-supportive items loading on one (empowering factor) and ego-involving and controlling items loading on a second (disempowering) factor. The findings of the current study are consistent with aforementioned [40] offering support for a two lower-order factor structure for the EDMCQ-C in Lithuanian youth sport participants. It should be noted that across groups two and three, a number of disempowering items loaded significantly onto the empowering factor (item 5 “My coach substituted players when they made a mistake,” item 15 “My coach only allows something we like to do at the end of training if players have done well during the session” and item 29 “The coach mainly used rewards/praise to make players complete all the tasks he/she sets during training”). Likewise, in group three, a small number of empowering items also loaded significantly onto the disempowering factor (item 13 “My coach acknowledged players who tried hard” and item 22 “When my coach asked players to do something, he or she tried to explain why this would be good to do so”). However, given the factor loading for these items on the non-intended factor were small and lower compared to the factor loadings on their intended factor and that cross-loading items are permitted (and expected) within ESEM analyses, we recommend these items are retained in the Lithuania version of the EDMCQ-C with the caveat that researchers re-examine the factor loading in their own data sets in future research using the scale.

### 4.2. Reliability of the EDMCQ-C

To determine the reliability of the Lithuania version of the EDMCQ-C, the internal consistency of athletes’ scores on the empowering and disempowering subscales were examined. The Cronbach’s alpha coefficients were satisfactorily high for both empowering and disempowering climates across samples two and three. In the initial validation [39] and subsequent studies [15], Cronbach’s alpha internal consistency reliability coefficients were 0.87 and 0.86. The higher alpha coefficients in our study revealed that the translated EDMCQ-C is a reliable measure of Lithuania athletes’ perceptions of the coach-created motivational climate. In addition, the conceptual model proposed by Duda [18] suggest that empowering and disempowering dimensions are distinct construct and thus we expected a weak negative but significant relationship between the two climate dimensions. Discriminant validity of the EDMCQ-Q was supported by a negative but not too strong, correlation among empowering and disempowering subscales.

### 4.3. Discriminant and Convergent Validity of the EDMCQ-C

Convergent validity was also assessed via associations between the empowering and disempowering subscales with expected correlates of the coach-created motivational climate. Evidence of convergent validity was established via positive correlations between empowering climate scores and athletes’ moral and competence values, intrinsic motivation and self-esteem and a negative association with amotivation. Conversely, disempowering climate scores were negatively associated with athletes’ self-esteem but positively correlated with athletes’ status value and amotivation. Regarding predictive validity, empowering but not disempowering climate scores emerged as a significant positive predictor of athletes’ self-reported prosocial behaviour in sport and disempowering but not empowering climates scores significantly and positively predicted athletes’ antisocial behaviour in sport. These findings build upon previous research [15,43,69] that has identified correlates of empowering and disempowering coach-created motivational climates and provides further evidence for the criterion validity of the EDMCQ-C and specifically the Lithuania version.

### 4.4. Predictive Validity of the EDMCQ-C

The aforementioned findings concerning criterion validity are explained by Duda and colleagues’ conceptual model of empowering and disempowering motivational climates [21,46] with specific reference to key assumptions within SDT and AGT. That is, Duda et al.’s model proposes that an empowering climate will be positively associated and predict outcomes such as prosocial moral values and behaviour, intrinsic motivation and self-esteem and be negatively associated with outcomes such as amotivation because this motivational environment is more likely to foster athletes’ feelings of autonomy, belonging and competence which are considered within SDT to be psychological nutrients required for optimal human functioning. Drawing from AGT, Duda et al.’s model also proposes that empowering climates will encourage athletes to adopt task-involving perceptions of competence which previous research has confirmed leads to adaptive cognition, affect and behaviour in sport. Conversely, Duda’s model accounts for the positive link between disempowering coaching strategies and amotivation and antisocial values and behaviour because this motivational climate is more likely to frustrate and/or lead low levels of autonomy, belonging and competence, as well as a more ego-involving view of competence which is a well-established predictor of maladaptive functioning in sport. Future research is now needed to test these theoretical assumptions regarding the psychological mechanisms that may account for the effects of empowering and disempowering climates in Lithuanian sport.

### 4.5. Limitations and Future Research Directions

Building upon the initial research that has tested the psychometrics of the EDMCQ [e. g., 39,40], we tested a range of validity and reliability indicators of the EDMCQ translated into Lithuanian. Given that establishing the psychometrics of this scale (and specifically in Lithuania) is still in its infancy, future research should consider additional forms of validity and reliability (e.g., test-re-test). We were also unable to account for the multilevel nature of the data in this study because of the limited number of units (i.e., teams) per parameter tested and because it is not currently possible to conduct a multilevel analysis in ESEM. Future research may overcome these limitations by collecting data from a larger number of teams and subsequently employing the “cluster” command in Mplus when testing the ESEM model [70]. Although this study included recreational junior athletes from a number (four) of sports, future research should continue to examine the psychometrics of the EDMCQ with a more heterogeneous sample of Lithuanian athletes. In addition to a wider range of individual and team sports, future research could test the psychometrics of the Lithuanian version of the EDMCQ with elite athletes and adult sport participants.

## 5. Conclusions

In summary, the purpose of the current research was to report the initial psychometric properties of the EDMCQ when translated into Lithuanian and completed by junior sport participants. The evidence from this study is aligned with the findings of previous research (e.g., [40]) that supports a two-factor lower-order model. Moreover, the research provides additional evidence regarding indicators of validity and reliability of the scale. This evidence suggests the EDMCQ-C(LT) can be used in future research to examine the antecedent and consequences of junior athletes’ perceptions of empowering and disempowering features of the coach-created motivational climate in Lithuanian sport.

## Figures and Tables

**Table 1:** Demographic characteristics of the study sample, N = 712.

Characteristics	Mean (SD) or Frequency (%)
Gender Male Female	458 (64.3) 254 (35.7)
Age	14.42 (1.08)
Sport experience (No of seasons)	4.39 (2.49)
Current sport	
Basketball	277 (38.9)
Volleyball	216 (15.7)
Football	112 (30.3)
Track and field	107 (15.1)

**Table 2:** Items, standardized factor loadings (FL) for Empowering and Disempowering Motivational Climate Questionnaire-Coach (EDMCQ-C) (group two).

EDMCQ-C Items	Factor loading		M (SD)	Skewness	Kurtosis
	DC	EC			
9. My coach gave most attention to the best players	0.563 ***	−0.117	2.75 (1.12)	0.43	0.52
12. My coach paid less attention to players if they displeased him or her	0.635 ***	−0.195 *	1.91 (0.94)	0.82	0.26
33. My coach favoured some players more than others	0.692 ***	−0.072	2.75 (1.05)	0.22	−0.42
24. My coach shouts at players in front of others to make them do certain things	0.705 ***	0.150 *	2.55 (1.25)	0.37	−0.82
17. My coach was less accepting of players if they disappointed him or her	0.776 ***	−0.093	2.50 (0.95)	0.21	−0.17
20. My coach only rewards players with prizes or treats if they have played well	0.655 ***	−0.012	2.51 (0.99)	0.37	−0.21
7. My coach was less supportive of players when they were not training and/or playing well	0.717 ***	−0.003	2.90 (1.14)	−0.07	0.91
19. My coach had his or her favourite players	0.516 ***	0.177 *	3.27 (1.05)	−0.18	0.41
26. My coach threatened to punish players to keep them in line during training	0.620 ***	0.050	2.56 (1.20)	0.18	−1.01
21. My coach only praised players who performed the best during a match	0.502 ***	0.002	2.81 (1.11)	0.21	−0.73
10. My coach yelled at players for messing up	0.639 ***	0.015	2.59 (1.18)	0.31	−0.80
5. My coach substituted players when they made a mistake	0.372 ***	0.181 *	3.31 (1.25)	−1.07	0.23
2. My coach was less friendly with players if they didn’t make the effort to see things his/her way	0.610 ***	−0.092	2.78 (1.19)	0.57	−1.03
25. My coach thought that only the best players should play in a match	0.590 ***	0.004	2.76 (1.13)	0.22	−0.68
31. My coach tried to interfere in aspects of players’ lives outside of this sport	0.636 ***	0.046	2.17 (1.15)	0.79	−0.23
15. My coach only allows something we like to do at the end of training if players have done well during the session	0.558 ***	−0.046	2.74 (0.92)	−0.32	1.01
29. The coach mainly used rewards/praise to make players complete all the tasks he/she sets during training	0.737 ***	0.115 *	2.71 (0.87)	0.52	0.87
23. My coach made sure everyone had an important role on the team	−0.077	0.824 ***	4.43 (0.71)	−1.08	0.75
8. My coach could really be counted on to care, no matter what happened	0.020	0.715 ***	4.51 (0.72)	−0.84	0.65
11. My coach made sure players felt successful when they improved	0.056	0.803 ***	4.36 (0.87)	−0.79	0.22
28. My coach let us know that all the players are part of the team’s success	−0.047	0.549 ***	4.31 (0.86)	−1.26	1.03
14. My coach really appreciated players as people, not just as athletes	−0.144 *	0.474 ***	4.37 (0.75)	−1.07	1.02
16. My coach answered players’ questions fully and carefully	−0.067	0.742 ***	4.53 (0.61)	−0.91	0.15
4. My coach tried to make sure players felt good when they tried their best	0.028	0.712 ***	4.57 (0.65)	−1.05	0.92
22. When my coach asked players to do something, he or she tried to explain why this would be good to do so	0.110	0.540 ***	4.32 (0.78)	−1.19	0.71
30. My coach encouraged players to help each other learn	−0.077	0.548 ***	4.20 (0.83)	−0.75	−0.16
3. My coach gave players choices and options	−0.038	0.533 ***	4.17 (0.86)	−1.05	0.91
1. My coach encouraged players to try new skills	0.001	0.580 ***	4.37 (0.79)	−0.51	0.92
34. My coach encouraged players to really work together as a team	−0.121	0.571 ***	4.42 (0.90)	−1.08	0.51
18. My coach made sure that each player contributed in some important way	0.018	0.812 ***	4.37 (0.86)	−0.67	0.10
27. My coach listened openly and did not judge players’ personal feelings	−0.152 *	0.509 ***	4.24 (0.84)	−1.08	0.55
13. My coach acknowledged players who tried hard	0.168 *	0.517 ***	4.31 (0.72)	−1.08	0.93
6. My coach thought that it is important that players participate in this sport because the players really want to	−0.091	0.629 ***	4.28 (0.70)	−0.79	0.70
32. My coach thought that it is important for players to play this sport because they (the players) enjoy it	0.013	0.542***	4.00 (0.85)	−0.63	0.12

DC = Disempowering climate, EC = Empowering climate. * *p* < 0.05, *** *p* < 0.001.

**Table 3:** Items, standardized factor loadings (FL) for EDMCQ-C re-test (group three).

EDMCQ-C Items	Factor Loading		M (SD)	Skewness	Kurtosis
	DC	EC			
9	0.646 **	−0.179 ***	2.63 (1.15)	0.44	−0.54
12	0.573 ***	−0.306 ***	1.90 (0.98)	0.89	0.14
33	0.634 ***	−0.190 ***	2.57 (1.10)	0.31	−0.51
24	0.713 ***	0.072	2.49 (1.26)	0.39	−0.90
17	0.750 ***	−0.091	2.47 (1.02)	0.30	−0.37
20	0.591 ***	−0.013 *	2.52 (1.10)	0.23	−0.74
7	0.704 ***	0.001	2.85 (1.16)	−0.04	−0.91
19	0.601 ***	−0.005	3.07 (1.09)	−0.18	−0.52
26	0.659 ***	0.063	2.44 (1.24)	0.37	−1.01
21	0.564 ***	0.010	2.82 (1.18)	0.15	−0.92
10	0.681 ***	0.020	2.57 (1.20)	0.30	−0.91
5	0.426 ***	0.123 **	3.26 (1.18)	0.20	−1.07
2	0.611 ***	−0.080	2.65 (1.21)	0.21	−1.01
25	0.626 ***	0.013	2.62 (1.17)	0.35	−0.69
31	0.598 ***	−0.012	2.10 (1.14)	0.90	0.03
15	0.398 ***	0.131 **	3.06 (1.10)	−0.01	−0.54
29	0.357 **	0.163 ***	2.98 (1.03)	−0.36	−0.52
23	−0.061	0.781 ***	4.36 (0.78)	−1.03	0.20
8	−0.041	0.723 ***	4.49 (0.72)	−1.09	0.21
11	0.083	0.762 ***	4.35 (0.70)	−0.98	0.97
28	−0.031	0.547 ***	4.30 (0.87)	−1.08	0.45
14	−0.069	0.584 ***	4.28 (0.81)	−1.10	0.81
16	0.010	0.762 ***	4.50 (0.68)	−1.08	0.93
4	−0.036	0.736 ***	4.52 (0.69)	−1.05	0.95
22	0.168 **	0.581 ***	4.15 (0.89)	−1.15	1.06
30	−0.117 *	0.579 ***	4.29 (0.80)	−1.16	0.65
3	−0.001	0.509 ***	4.24 (0.82)	−1.08	0.93
1	0.062	0.631 ***	4.35 (0.80)	−1.06	0.96
34	−0.007	0.623 ***	4.44 (0.83)	−1.25	0.77
18	0.109	0.732 ***	4.30 (0.72)	−0.89	1.05
27	−0.162 **	0.519 ***	4.13 (0.93)	−1.07	0.70
13	0.119 **	0.585 ***	4.30 (0.81)	−1.23	0.83
6	−0.015	0.689 ***	4.25 (0.75)	−0.86	0.63
32	0.075	0.580 ***	4.03 (0.84)	−0.67	0.28

DC = Disempowering climate, EC = Empowering climate. * *p* < 0.05, ** *p* < 0.01, *** *p* < 0.001.

**Table 4:** Correlations between EDMCQ-C subscales and values, motivation in sport and self-esteem variables.

Variables	1	2	3	4	5	8	9	10
1. EC								
2. DC	−0.23 **							
3. Moral	0.40 **	−0.05						
4. Competence	0.34 **	−0.03	0.62**					
5. Status	0.19 **	0.16 **	0.27**	0.49 **				
8. Intrinsic	0.49 **	0.03	0.32**	0.52 **	0.35 **			
9. Amotivation	−0.30 **	0.32 **	−0.19**	−0.29 **	−0.06	−0.35 **	0.01	
10. Self-esteem	0.22 **	−0.29 **	0.18**	0.18 **	0.13 *	0.24 **	0.19 **	−0.31 **

EC = Empowering climate, DC = Disempowering climate. Values in bold with greater interest. * *p* < 0.05, ** *p* < 0.01

**Table 5:** The predictive ability of EDMCQ-C in relation to athletes’ moral behaviour in sport.

Variable	B	*B* 95% CI	*β*	*t*	Δ*R*^2^
Prosocial behaviour					
Empowering climate	0.43	0.27< >0.59	0.26	5.17 ***	
Disempowering climate	0.07	−0.04< >0.19	0.06	*ns*	
					0.06 ***
Antisocial behaviour					
Empowering climate	−0.03	−0.14< >0.09	−0.02	*ns*	
Disempowering climate	0.23	0.16< >0.31	0.30	5.28 ***	
					0.09 ***

A*R*^2^ = R^2^ unique to each step, *** *p* < 0.001, CI = confidence interval, *ns* = not significant.

## Supplementary Materials

The following are available online at https://www.mdpi.com/1660-4601/17/10/3487/s1.
