# Supplementary material for: Validation of the Lithuanian Version of the Coach-Created Empowering and Disempowering Motivational Climate Questionnaire (EDMCQ-C)
Source: Int J Environ Res Public Health. 2020 May 16;17(10):3487. doi: 10.3390/ijerph17103487 (PMC7277585; doi:10.3390/ijerph17103487)
Supplement: Supplementary file 1 [file ijerph-17-03487-s001.pdf]

Lithuanian version of the EDMCQ-C items

1. Mano treneris skatino sportininkus išbandyti naujus gebėjimus
2. Mano treneris buvo mažiau draugiškas sportininkams, jei jie ne taip atkakliai stengėsi, kaip jis norėjo
3. Mano treneris leido sportininkams rinktis ar išsakyti savo nuomonę
4. Mano treneris stengėsi, jog sportininkai jaustųsi gerai, kai stengėsi iš visų jėgų
5. Mano treneris pakeisdavo sportininkus, jei jie darydavo klaidas
6. Mano treneris manė, kad sportininkai sportuoja, nes jie tikrai to nori
7. Mano treneris buvo ne toks geranoriškas sportininkams, kai jie nepakankamai gerai treniruodavosi ar rungtyniaudavo
8. Iš mano trenerio buvo galima tikėtis pagalbos bet kurioje situacijoje
9. Mano treneris daugiausia dėmesio skyrė geriausiems sportininkams
10. Mano treneris apšaukdavo sportininkus, kurie buvo netvarkingi
11. Mano treneris skatino sportininkus jaustis sėkmingais, kai jie patobulėjo
12. Mano treneris skyrė mažiau dėmesio sportininkams, kurie jam nepatikdavo
13. Mano treneris pripažindavo /pastebėdavo tuos sportininkus, kurie labai stengėsi
14. Mano treneris tikrai vertino kiekvieną žaidėją kaip žmogų, o ne tik kaip sportininką
15. Tik tuomet, kai visos treniruotės metu gerai dirbdavome, treneris jos pabaigoje leisdavo daryti tai, kas mums patinka
16. Treneris išsamiai ir pilnai atsakydavo į sportininkų klausimus
17. Mano treneris buvo mažiau geranoriškas sportininkams, kurie jį nuvildavo
18. Mano treneris užtikrindavo, kad kiekvienas sportininkas rimtai dirbtų
19. Mano treneris turėjo savo mėgstamus sportininkus
20. Mano treneris apdovanodavo tik tuos sportininkus, kurie gerai rungtyniaudavo
21. Mano treneris pagirdavo tik geriausiai rungtynių metu pasirodžiusius sportininkus
22. Kai mano treneris duodavo užduotis sportininkams, jis stengdavosi paaiškinti kam to reikia
23. Mano treneris stengėsi, jog visi sportininkai jaustųsi esantys svarbūs komandoje
24. Mano treneris aprėkdavo sportininkus kitų sportininkų akivaizdoje, kad priverstų juos atlikti konkrečias užduotis
25. Mano treneris mano, kad tik geriausi sportininkai gali žaisti rungtynių metu
26. Mano treneris grasino nubausti sportininkus, jei jie nesilaikys drausmės treniruotėje
27. Mano treneris buvo atidus visų atžvilgiu ir nesmerkdavo sportininkų asmeninių jausmų
28. Mano treneris leido mums suprasti, kad nu visų kartu priklauso komandos sėkmė.
29. Mano treneris įvairiais pagyrimais ir apdovanojimais skatindavo sportininkus atlikti jiems skirtas užduotis
30. Mano treneris skatino sportininkus vienas kitam padėti.
31. Mano treneris bandė kištis į sportininkų asmeninį gyvenimą už sporto ribų
32. Mano treneris manė, kad sportininkams svarbu sportuoti pasirinktą sporto šaką, nes ji jiems patinka
33. Mano treneriui vieni sportininkai patikdavo labiau, nei kiti
34. Mano treneris skatino sportininkus dirbti kartu kaip komanda
